# Surgical site infection after laparoscopic resection of colorectal cancer is associated with compromised long-term oncological outcome

**DOI:** 10.1186/s12957-022-02578-2

**Published:** 2022-04-07

**Authors:** Nana Sugamata, Takashi Okuyama, Emiko Takeshita, Haruka Oi, Yuhei Hakozaki, Shunya Miyazaki, Musashi Takada, Takashi Mitsui, Takuji Noro, Hideyuki Yoshitomi, Masatoshi Oya

**Affiliations:** Department of Surgery, Dokkyo Medical University Saitama Medical Center, 2-1-50 Minami-Koshigaya, Koshigaya, Saitama, 343-8555 Japan

**Keywords:** Surgical site infection, Colorectal cancer, Laparoscopic surgery, Postoperative infection

## Abstract

**Background:**

We evaluated the influence of infectious complications, particularly surgical site infection (SSI), on long-term oncological results after elective laparoscopic resection of colorectal cancer.

**Methods:**

A total of 199 patients who underwent laparoscopic elective resection with negative resection margins for stage I–III colorectal cancer were retrospectively examined. The postoperative course was recorded based on hospital records, and cancer relapse was diagnosed based on radiological or pathological findings under a standardized follow-up program. The severity of complications was graded using Clavien-Dindo (CD) classification.

**Results:**

SSI was found in 25 patients (12.6%), with 12 (6.0%) showing anastomotic leak. The postoperative relapse-free survival (RFS) rate was significantly lower in patients with SSI (49.2%) than in patients without SSI (87.2%, *P*<0.001). Differences in RFS were found after both colectomy and rectal resection (*P*<0.001 and *P*<0.001, respectively). RFS did not differ between patients who had major SSI CD (grade III) and those who had minor SSI CD (grades I or II). Multivariate Cox regression analysis identified the occurrence of SSI and pathological stage as independent co-factors for RFS (*P*<0.001 and *P*=0.003).

**Conclusion:**

These results suggest that postoperative SSI compromises long-term oncological results after laparoscopic colorectal resection. Further improvements in surgical technique and refinements in perioperative care may improve long-term oncological results.

## Introduction

Laparoscopic surgery has commonly been applied for colorectal cancer in recent years. Postoperative complications are reportedly less frequent after laparoscopic surgery than after open surgery [1–3]. Long-term oncological results have been shown to be comparable between laparoscopic and open surgeries [4–6].

Infectious complications after surgery have been reported to compromise long-term oncological results in gastric cancer [7–9], esophageal cancer [10], and breast cancer [11]. With regard to colorectal cancer, although anastomotic leak (AL) has been suggested as a risk factor for local recurrence [12–15], the impact of infectious complications including surgical site infections (SSIs) such as AL on long-term oncological outcome after resection of colorectal cancer has been controversial [12–24]. In particular, studies limited to patients after laparoscopic colorectal resection remain scarce [25]. The present study explored the relationship between SSI and long-term oncological results in patients who underwent laparoscopic curative resection for stage I–III colorectal cancer in our surgical department.

## Patients and methods

A total of 405 patients underwent elective resection for colorectal cancer between January 2013 and May 2015 in the Department of Surgery at Saitama Medical Center, Dokkyo Medical University. Of those 405 patients, 239 patients underwent laparoscopic resection. Fifteen patients with pathological pT0 lesions after neoadjuvant treatment or pTis lesion and 14 patients with clinically confirmed stage IV lesion were excluded. The tumor stage was based on the classification by the Japanese Society for Cancers of the Colon and Rectum [26]. Eleven patients with positive or unclear resection margins were also excluded. As a result, a total of 199 patients were included in the study.

Diabetes mellitus (DM) which is a well-established risk factor for colorectal cancer was recorded as present in patients receiving oral medication or insulin injection or in those with hemoglobin (Hb)A1c level ≥6.5% without treatment for DM [27]. Systemic co-morbidities including DM were evaluated using the American Society of Anesthesiology physical status. The surgical procedures performed were colectomy including anterior resection for rectosigmoid cancer in 138 patients and rectal resection in 61 patients. Adjuvant chemotherapy was carried out mainly in stage III patients for 6 months using a regimen of oral 5-fluorouracil-leucovorin or capecitabine alone, or regimens including oxaliplatin (FOLFOX or CAPOX). Neoadjuvant treatment including neoadjuvant chemoradiotherapy was performed mainly for patients with rectal cancer.

Patients received mechanical bowel preparation comprising magnesium citrate solution and laxatives the day before surgery. Preoperative oral antibiotics were not applied. Prophylactic intravenous antibiotics were administered at the time of skin incision and every 4 h thereafter during surgery, in the evening of the day of surgery, and twice on the first postoperative day.

The duration of operation and intraoperative bleeding was recorded from the hospital records, which were completed by residents, ward nurses, and attending surgeons. Perioperative morbidity up to 30 days after surgery was also retrieved by two of the authors (NS and MO) from the hospital records. Infectious complications were recorded mainly based on clinical symptoms such as fever, pain and tenderness, radiological findings, and urinalysis. SSI was defined according to the Centers for Disease Control and Prevention (CDC) guidelines [28]. The severity of postoperative complications was graded according to Clavien-Dindo (CD) classification [29].

Postoperative follow-up was standardized as follows: blood testing, including measurement of carcinoembryonic antigen (CEA) every 3 months; and CT from the chest to the pelvis every 6 months until 5 years after surgery. Surveillance colonoscopy was usually performed within 1 year after surgery and annually thereafter until no neoplastic lesion was detected. If no neoplastic lesions were detected on surveillance colonoscopy, the next colonoscopy was performed 3 years later. When CEA concentration was elevated without accompanying CT, CT was usually performed. Relapse was diagnosed based on the findings from CT or biopsy at colonoscopy. Positron-emission tomography (PET) was occasionally performed to confirm relapse.

Relapse-free survival (RFS) time was calculated as the duration between surgery and diagnosis of relapse. If a patient died without a diagnosis of relapse, patient data were censored as of the time of death. Overall survival (OS) was also recorded as the duration between surgery and death from any cause, including other causes than colorectal cancer. However, since OS is strongly affected by the treatment after relapse, OS was not analyzed as an endpoint of the present study. The median duration of follow-up for RFS was 60.8 months.

### Statistical analysis

Postoperative RFS was compared between patients who did and did not develop infectious complications using Kaplan-Meier methods with log-rank testing. The influence of various factors including SSI on RFS was examined using uni- and multivariate Cox proportional hazard regression modeling. Multivariate analysis was performed using a forward stepwise selection method. Factors that trended toward significance (*P*<.10) on univariate analysis were included as candidates for independent variables affecting disease-free survival (DFS). A two-sided value of *P* <.05 was considered significant. All statistical analyses were performed using the Dr. SPSS software package (SPSS Japan, Tokyo, Japan).

## Results

### Postoperative complications

Patient characteristics and the details of adjuvant chemotherapy and neoadjuvant treatment are shown in Tables 1 and 2. Postoperative SSIs in a total of 199 patients are summarized in Table 3. SSI occurred in 25 patients (12.6%, Table 4), including in 14 patients (10.1%) after colectomy and 11 patients (18.0%) after rectal resection. Of the 25 patients who developed SSI, 18 patients showed minor SSI (CD grades I or II), whereas 7 patients developed major SSI (grades III). Major SSI occurred in only 1 patient after colectomy and 6 patients after rectal resection. Although AL occurred in 4 patients after colectomy and 8 patients after rectal resection, CD grades were II in 7 patients and III in 5 patients. No postoperative deaths occurred within 90 days after surgery.

**Table 1:** Patients’ characteristics

Patients’ characteristics	Number (%)
Age^a^	69.3 (62.6–74.7)
Gender	
Male	124 (62.3%)
Female	75 (37.8%)
Body mass index (BMI)	
<18.5	22 (11.1%)
18.5 ≤ 25	145 (72.9%)
25−	32 (16.0%)
Physical status of American Society of Anesthesiology	
1	113 (56.8%)
2	80 (40.2%)
3	6 (3.0%)
Diabetes mellitus (DM)	
Absent	159 (79.9%)
Present	40 (20.1%)
Operative procedures	
Colectomy	138 (69.3%)
Ileocecal resection	14 (10.1%)
Right hemicolectomy (RHC)	18 (13.0%)
Transverse colectomy	2 (1.4%)
Left hemicolectomy	16 (11.6%)
Sigmoidectomy (SC)	50 (36.2%)
Anterior resection	33 (23.9%)
RHC+SC	2 (1.4%)
SC+total gastrectomy	1 (0.7%)
Total colectomy	2 (1.4%)
Rectal resection	61 (30.7%)
Low anterior resection	27 (44.3%)
Very low anterior resection	24 (39.3%)
Abdominoperineal resection	10 (16.4%)
Duration of operation^a^	217 (180–280)
Intraoperative bleeding^a^	20 (10–50)
Pathological stage	
I	59 (29.6%)
II	71 (35.7%)
III	69 (34.7%)
Preoperative CEA	
>5ng/ml	139 (69.8%)
≦5ng/ml	63 (30.2%)
Preoperative CA19-9	
>37ng/ml	161 (80.9%)
≦37ng/ml	38 (19.1%)

^a^Values show median with interquartile ranges in parentheses

**Table 2:** Perioperative treatment according to pathological stage

	Stage I	Stage II	Stage III
Neoadjuvant treatment			
No	56 (94.9%)	61 (85.9%)	67 (97.1%)
Chemoradiotherapy	0	3 (4.2%)	2 (2.9%)
Chemotherapy	3 (5.1%)	7 (9.9%)	0
Adjuvant treatment			
No	59 (100%)	64 (90.1%)	19 (27.5%)
Oral chemotherapy	0	6 (8.5%)	22 (31.9%)
Intravenous chemotherapy	0	1 (1.4%)	28 (40.6%)

Patients who received neoadjuvant chemoradiotherapy or systemic chemotherapy had lymph node metastasis less frequently than those who did not.

**Table 3:** Postoperative surgical site infections

Intra-abdominal infection	14 (7.0%)
Anastomotic leak	12 (6.0%)
Intra-abdominal abscess	2 (1.0%)
Other surgical infection	11 (5.5%)
Perineal wound infection	3 (1.5%)
Other wound infection	6 (3.0%)
Infection at the drain site	1 (0.5)
Peristomal infection	1 (0.5%)

**Table 4:** The difference in patients' background according to the presence of postoperative SSI

	SSI+	SSI-	*P*=
Gender			0.66
Male	17 (68.0%)	107 (61.5%)	
Female	8 (32.0%)	67 (38.5%)	
Age (years)*	66.5 (57.2–74.3)	69.5 (63.0–74.7)	0.28†
Operative procedure			0.16
Colectony	14 (56.0%)	124 (71.3%)	
Rectal resection	11 (44.0%)	50 (28.7%)	
Body mass index			
Normal	4 (16.0%)	18 (10.3%)	0.3
Intermediate	15 (60.0%)	130 (74.7%)	
High	6 (24.0%)	15 (15.0%)	
Diabetes mellitus			0.79
Normal	36 (90.0%)	138 (86.8%)	
Abnormality	4 (10.0%)	21 (13.2%)	
ASA			0.28
I	16 (64.0%)	97 (55.7%)	
II	8 (32.0%)	72 (41.4%)	
III	1 (4.0%)	5 (2.9%)	
Operating time (min)*	263 (182–328)	216 (180–280)	0.16^†^
Intraoperative bleeding (ml)*	20 (7.5–11.5)	20 (10–50)	0.58^†^
pStage			
I	4 (16.0%)	55 (31.6%)	0.21
II	11 (44.0%)	60 (34.5%)	
III	10 (40.0%)	59 (33.9%)	

The chi-square test, ^†^Mann-Whitney *U*

*Values show medians with interquartile ranges in parentheses

### Comparisons of RFS according to infectious complications

**Fig. 1:**
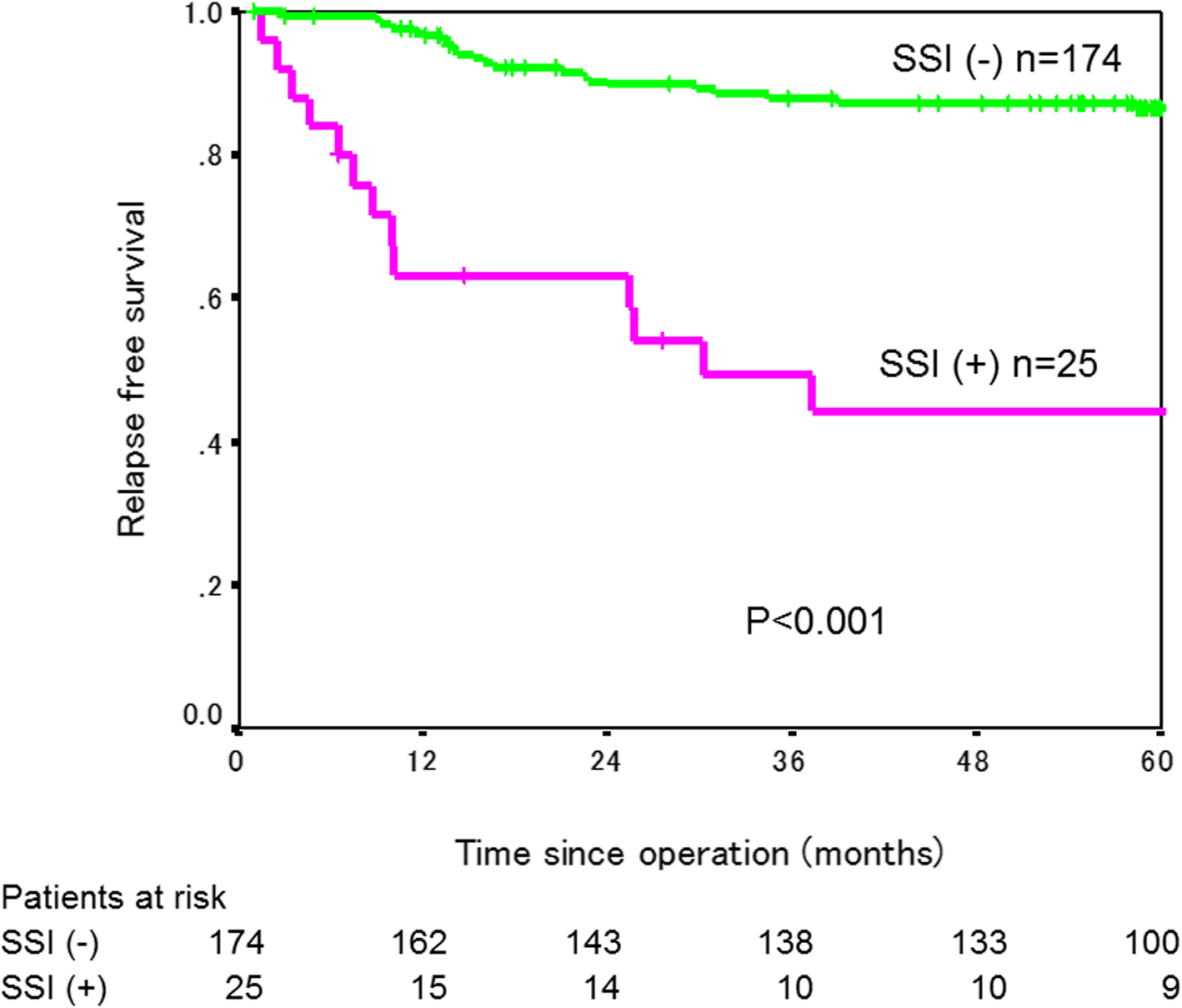
Relapse-free survival curves in patients with and without SSI

**Fig. 2:**
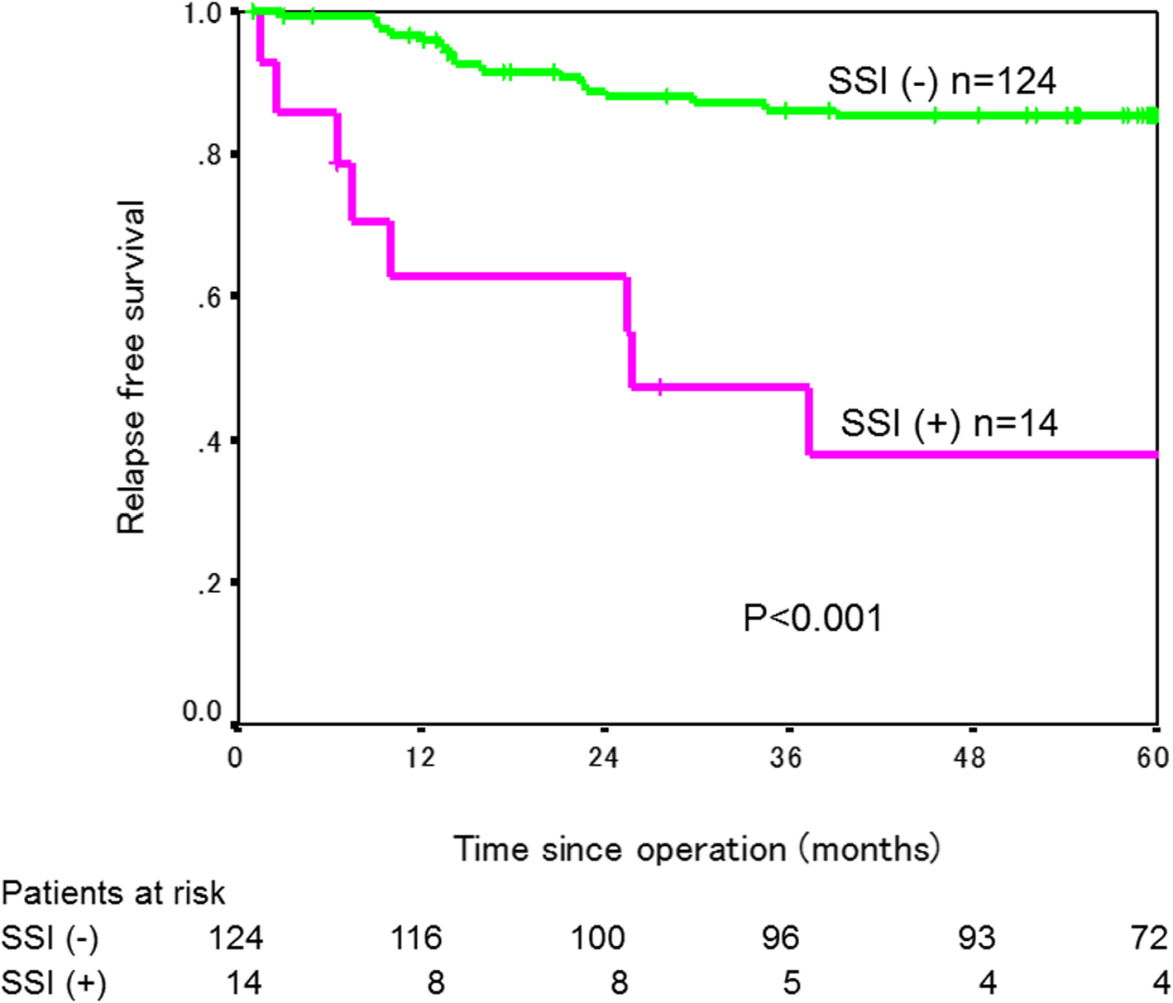
Relapse-free survival curves in patients with and without SSI after colectomy

**Fig. 3:**
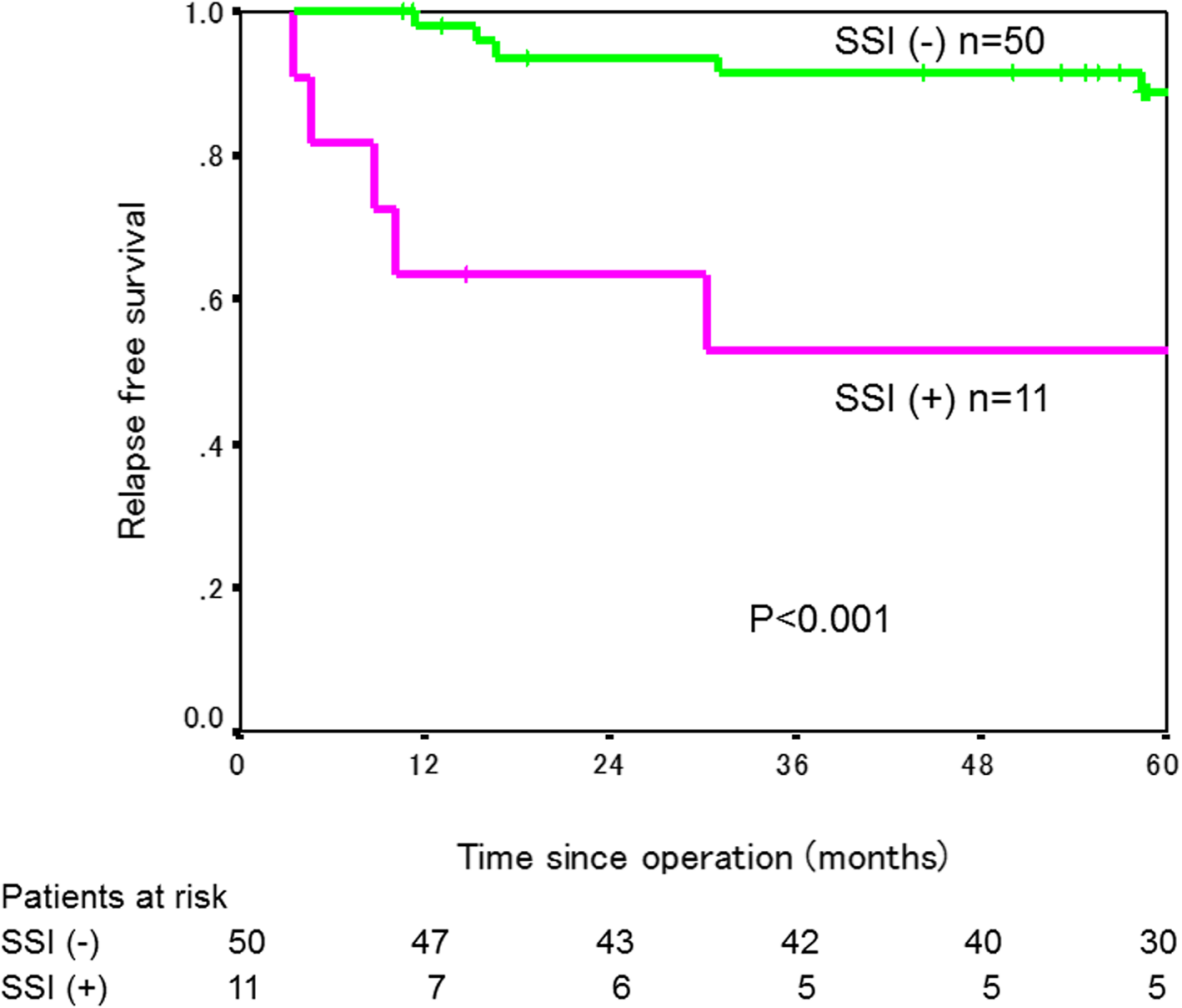
Relapse-free survival curves in patients with and without SSI after rectal resection

**Fig. 4:**
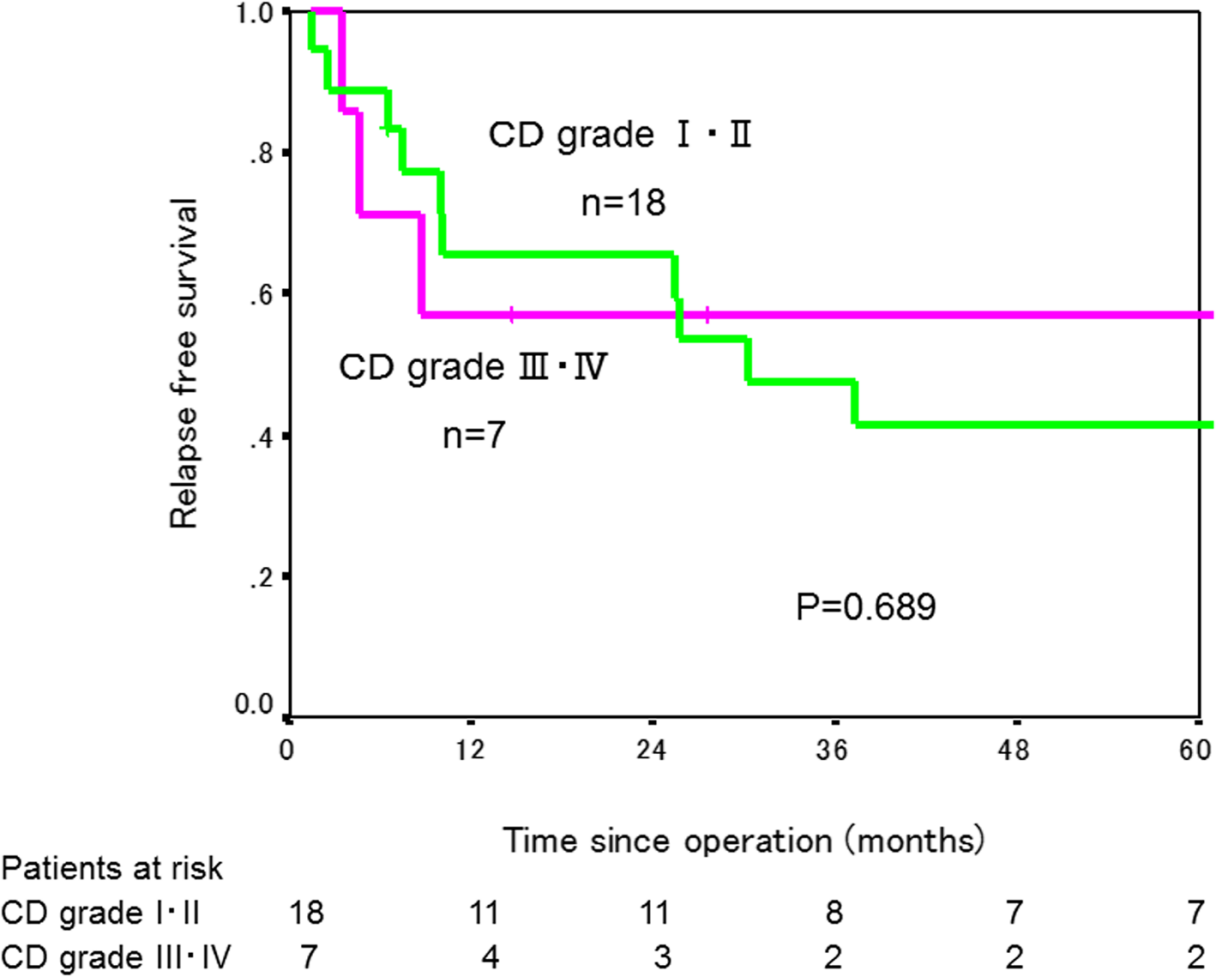
Relapse-free survival curves in patients who had major SSI with Clavien-Dindo (CD) grade I or II and in those who had minor SSI with CD grade III or IV

The 3-year RFS rate was significantly lower in the 25 patients with SSI (49.2%) than in the 174 patients without SSI (87.2%, *P*<0.001, Fig. 1). When patients after colectomy and patients after rectal resection were analyzed separately, a 3-year RFS rate was significantly worse in patients who developed SSI than in those who did not in both colectomy and rectal resection (colectomy: 47.1% in 14 patients with SSI versus 86.3% in 124 patients without SSI, *P*<0.001; rectal resection: 53.0% in 11 patients with SSI versus 91.4% in 50 patients without SSI, *P*<0.001, Figs. 2 and 3). RFS rate did not differ between patients with major SSI (CD grade III/IV) and those with minor SSI (CD grade I/II) (*P*=0.689, Fig. 4).

### Factors associated with RFS

Table 5 shows the results of univariate Cox proportional regression modeling for RFS. SSI was associated with shorter RFS. Other factors associated with shorter RFS were pathological stage II/III compared with stage I and intravenous adjuvant chemotherapy. In multivariate Cox proportional regression modeling, pathological stage and the presence of SSI were significantly associated with shorter RFS (Table 6). In the sub-analysis by stage, stage I cases showed no significant difference in a 3-year RFS rate according to the occurrence of SSI (SSI− vs. SSI+, in stage I: 100 vs. 100%). By contrast, significant differences in a 3-year RFS rate were seen in each analysis for stage II and stage III (SSI− vs. SSI+, in stage II: 53.3 vs. 89.2%, *P*=0.0001; in stage III: 26.7 vs. 75.4%, *P*<0.0001).

**Table 5:** Influence on relapse-free survival (univariate proportional hazard model)

		Hazard ratio	95% confidence interval		*P*=
Age			0.963	1.023	0.635
Gender					
Male		1			
Female		0.775	0.389	1.544	0.470
BMI					
<18.5		1			
18.5≤25		0.556	0.228	1.356	0.197
25−		0.652	0.210	2.024	0.460
ASA					
1		1			
2		1.094	0.560	2.137	0.792
3		2.899	0.670	12.539	0.154
Diabetes mellitus					
Absent		1			
Present		1.016	0.446	2.314	0.969
Operative procedures					
Colon		1			
Rectum		0.922	0.456	1.867	0.821
Duration of operation		0.997	0.993	1.001	0.155
Intraoperative bleeding		0.999	0.996	1.002	0.571
Pathological stage					
I		1			
II		11.926	1.560	91.190	0.017
III		22.984	3.103	170.239	0.002
Preoperative CEA					
>5ng/ml		1			
≦5ng/ml		1.580	0.812	3.071	0.177
Preoperative CA19-9					
>37ng/ml		1			
≦37ng/ml		1.206	0.551	2.640	0.639
Infectious complication					
Absent		1			
Present		3.743	1.924	7.284	<0.001
Surgical site infection					
Absent		1			
Present		6.206	3.185	12.095	<0.001
Preoperative adjuvant treatment					
No		1			
Chemoradiotherapy		0.924	0.222	3.852	0.914
Chemotherapy		1.120	0.153	8.186	0.911
Adjuvant chemotherapy					
No		1			
Oral		2.099	0.882	4.994	0.094
Intravenous		2.810	1.336	5.909	0.006

In the univariate proportional hazard model, pathological stage, infectious complication, and adjuvant intravenous chemotherapy were significantly associated with postoperative RFS.

**Table 6:** Result of multivariate proportional hazard model on relapse-free survival (forward stepwise selection method)

	Hazard ratio	95% confidence interval		*P*=
Pathological stage				
I	1			
II	9.919	1.293	76.071	0.027
III	20.758	2.799	153.967	0.003
Surgical site infection				
Absent	1			
Present	5.640	2.883	11.032	<0.001

In multivariate proportional hazard model, pathological stage and SSI were co-factors significantly associated with RFS

### Site of relapse

Table 7 compares sites of relapse according to the occurrence of SSI. Liver, distant lymph node, and peritoneal dissemination recurrences were significantly more frequent in patients with SSI compared to those without (*P*=0.009, *P*=0.04, and *P*=0.04, respectively). In patients without SSI, lung metastasis was the most frequent site of relapse. The local recurrence occurred in 4 patients, comprising 2 patients with SSI and 2 patients without.

**Table 7:** Site of recurrence according to the absence or presence of SSI

	SSI (−)		SSI (+)		*P*=
	Number of patients (%)	% of recurrent patients	Number of patients (%)	% of recurrent patients	
Total	174		25		
Recurrence (+)	23 (13.2%)		14 (56.0%)		
Local	2 (1.1%)	8.7%	2 (8.0%)	14.3%	0.07
Liver^a^	7 (4.0%)	30.4%	5 (20.0%)	35.7%	0.009
Lung	10 (5.7%)	43.5%	2 (8.0%)	14.3%	0.65
Lymph nodes^a^	4 (2.3%)	17.4%	3 (12.0%)	21.4%	0.04
Peritoneal^a^	4 (2.3%)	17.4%	3 (12.0%)	21.4%	0.04
Multiple sites	4 (2.3%)	17.4%	1 (4.0)	7.1%	0.49

*SSI* surgical site infection

^a^SSI (−) versus SSI (+) of all patients

### Adjuvant chemotherapy in patients with stage III lesion

Table 8 shows adjuvant chemotherapy in patients with stage III lesions. In 10 patients who had SSI, adjuvant chemotherapy was carried out in 5 patients (50%). By contrast, among the 59 patients who did not have SSI, adjuvant chemotherapy was performed in 45 patients (76.3%). The rate of patients who received adjuvant chemotherapy tended to be lower in patients with SSI than in patients without (*P*=0.09, chi-square test).

**Table 8:** Adjuvant chemotherapy in patients with pathological stage III lesion

	SSI	
	Absent	Present
No	14 (23.7%)	5 (8.5%)
Oral chemotherapy	21 (35.6%)	1 (1.7%)
Intravenous chemotherapy	24 (40.7%)	4 (6.8%)

*P*=0.142*

*χ^2^ test

## Discussion

The present study showed that postoperative SSI was significantly associated with inferior RFS after potentially curative laparoscopic resection for stage I–III colorectal cancer. The significance of the association was independent of the tumor stage and was present after both colonic resection and rectal resection.

Previous studies have usually included both patients after open surgery and those after laparoscopic surgery [14, 15, 19–24, 30–32]. However, laparoscopic surgery has been applied for the majority of elective resections for colorectal cancer in recent years. In our department, elective open surgery is selected on a limited basis for large lesions, those with suspected invasion into adjacent organs and those with extensive lymph node metastasis. These factors influence not only the pathological stage of the tumor, but also the technical difficulty of potentially curative resection. We therefore analyzed only those patients who underwent R0 resection by laparoscopic surgery in the present study.

The rates of AL after open rectal resection were around 10% in the literature [12, 13]. Akiyoshi et al. reported an AL rate of only 3.8% in 363 patients after laparoscopic anterior resection [33]. Others reported a lower incidence of SSI or LA after laparoscopic surgery than after open surgery [1–3]. In the present study, however, the LA rate in 51 patients after anterior resection for rectal cancer was 15.6% (8 of 51 patients, after excluding 10 patients who underwent abdominoperineal resection). The high incidence of AL after rectal resection in the present study may have been due to the affirmative application of very low anastomosis for low rectal lesions. The AL rate after colectomy (including anterior resection for rectosigmoid cancer) was low (only 4 of 138 patients, 2.9%). Moreover, AL in the present study was not always recorded as a major SSI (grade III or more in the CD classification).

SSI has been reported to be more frequent after rectal resection than after colectomy in both open surgery [34] and laparoscopic surgery [35]. In the present study, although SSI tended to be more frequent after rectal resection (11 of 61 patients) than after colectomy (14 of 138 patients), the difference between colectomy and rectal resection was not significant. This was probably due to the small number of patients.

Several previous studies, including meta-analyses, have shown that AL after rectal resection is a risk factor for local recurrence [12–15]. However, this association has not been found in other studies [18, 31]. In the present study, only one of the 12 patients who showed AL developed local recurrence. The overall incidence of local recurrence was low in the present study (only 4 of 199 patients, 2.0%).

The low incidence of local recurrence may be attributable to the exclusion from the present study of patients with positive or unclear resection margins. A positive circumferential resection margin is well established as a strong risk factor for local recurrence after mesorectal excision for rectal cancer [36]. For colonic cancer, recent studies have suggested the importance of negative resection margin for preventing local recurrence [37].

Reports exploring the impact of SSI on RFS or cancer-specific survival after laparoscopic surgery are limited. Park et al. reported that complications after laparoscopic low anterior resection for rectal cancer compromised RFS [25]. In the present study, the RFS rate was lower in patients with postoperative SSI than in those without, for both colectomy and rectal resection. Sub-analysis by stage found no significant difference in RFS rate according to the occurrence of SSI in patients with stage I, but a significant difference in patients with stages II or III. Further studies focused on these stages may support our results more strongly.

The impact of the severity of postoperative complications on long-term oncological results is still controversial. Odermatt et al. reported that increasing CD scores from grades I to IV was significantly associated with progressive decreases in OS and DFS [20]. In a report by Duraes et al., higher CD grades were associated with worse OS and RFS after colorectal cancer resection [21]. Cienfuegos et al. reported worse OS and DFS in patients with major complications beyond in CD grade IIIb than in those without major complications [22]. In contrast, Mark et al. reported similar oncological results after resection for rectal cancer among patients who had major complications, those who had minor complications and those without complications [19]. Oh et al. reported similar oncological results in patients with major (CD grade III or IV) or minor (grade I or II) complications after colorectal cancer surgery [24]. In the present study, RFS did not differ between patients who developed major SSI and those who developed minor SSI. The inclusion of only patients after laparoscopic surgery and stages I–III with negative resection margins might have influenced these results. In addition, the small number of patients with SSI might have been another reason for the negative result.

The mechanisms by which SSI results in worse RFS remain speculative. A possible explanation is that delayed or difficult postoperative adjuvant chemotherapy may be responsible for the inferior RFS in patients who had SSI [30]. Actually, the proportion of stage III patients in the present study who received adjuvant chemotherapy was marginally lower among patients who had SSI than among those who did not. However, this small difference in adjuvant chemotherapy may not sufficiently explain the significant difference in RFS between patients who did and did not show SSI.

Another issue for speculation is the negative influence of systemic or local inflammatory response caused by SSI on RFS. A number of studies have explored the influence of preoperative nutritional state and inflammation on oncological outcomes of various cancers [38–48]. Although we did not examine nutritional or inflammatory biomarkers in the present study, changes in such biomarkers caused by SSI may play a role in the long-term oncological outcomes. The relationship of perioperative nutritional or inflammatory biomarkers with long-term oncological results may be an interesting subject for future studies.

Although the present study found significant differences for some sites of recurrence (liver, lymph nodes, and peritoneum) between patients with and without SSI, the relationship between the site of recurrence and SSI is difficult to speculate. Inflammatory cells produce tumor necrosis factor α, transforming growth factor β, interleukin-6, and other cytokines, and these inflammatory agents regulate nuclear factor (NF)-κβ and the STAT3 pathway and encourage metastasis in tumor cells [49, 50]. Moreover, Helbig et al. reported that NFκβ induces the chemokine receptor CXCR4 which regulates organ-specific metastasis in several solid cancers and promotes cancer cell migration and metastasis [51]. These mechanisms should be investigated in future molecular studies.

The present study has some limitations that merit consideration when interpreting the results. First, this study included an inherent selection bias owing to its retrospective design. In particular, the selection bias was present with regard to selecting the surgical approach. In addition, co-morbidities other than DM might have affected the incidence of SSI and resulted in confounding bias. Second, the small number of patients makes it difficult to support the results conclusively. Third, this study was restricted to patients from a single institution. Details of the surgical technique for laparoscopic colorectal resection might differ between institutions, and different surgical techniques are likely to exert different influences on RFS, even among patients who had SSI. A multi-institutional survey of infectious complications including SSI and RFS using a unified protocol for the diagnosis of infectious complications and adjuvant chemotherapy may further clarify the influence of SSI on RFS.

In conclusion, the present study revealed that RFS after potentially curative laparoscopic resection of stage I–III colorectal cancer may be compromised by postoperative SSI. Further improvements in surgical technique and refinement of perioperative care to reduce SSI may contribute not only to the safety of colorectal surgery, but also to the improvement of long-term oncological results.

## Data Availability

The datasets supporting the conclusion of this article are included within the article. The underlying datasets are available from the corresponding author on reasonable request.
